# A new modality of treatment for non-united fracture of the humerus in a patient with osteopetrosis: a case report

**DOI:** 10.1186/1752-1947-3-15

**Published:** 2009-01-13

**Authors:** Imran Rafiq, Amit Kapoor, David JC Burton, John F Haines

**Affiliations:** 1 Upper Limb Unit, Wrightington Hospital, Hall Lane, Appley Bridge, Wigan, Lancashire, WN6 9EP, UK; 2 197, Berberis House, Highfield Road, Feltham, TW13 4GS, UK

## Abstract

**Introduction:**

Osteopetrosis introduces technical limitations to the traditional treatment of fracture management that may be minimised with specific pre-operative planning. Extreme care and caution are required when drilling, reaming, or inserting implants in patients with osteopetrosis. Caution must be exercised throughout the postoperative course when these patients are at greatest risk for device failure or further injury.

**Case presentation:**

We present our experience of treating such a fracture where a patient presented with a non-united fracture of the humerus. The bone was already osteoporotic. We successfully used a new technique which has not been described in the literature before. This included the use of a high-speed drill to prepare the bone for screw fixation. Bone healing was augmented with bone morphogenic protein.

**Conclusion:**

This technique can give invaluable experience to surgeons who are involved in treating these types of fracture.

## Introduction

Osteopetrosis is a rare skeletal condition first described by German radiologist Heinrich Albers-Schonberg in 1904 [1]. The condition is characterised by skeletal osteosclerosis caused by aberrant osteoclast-mediated bone resorption. Management of patients with osteopetrosis requires a comprehensive approach to characteristic clinical problems including metabolic abnormalities, fractures, deformities, back pain, bone pain, osteomyelitis and neurological sequelae [2]. Although fractures can be managed conservatively, they can be challenging when considering the internal fixation required to rectify non-union and mal-union. There have been many documented technical difficulties in operative management for fixation of fractures in these patients. We used a high-speed drill and bone morphogenic protein to treat a patient with a non-united fracture of the proximal humerus. We have not found any evidence of the use of this technique in the medical literature to treat fractures in osteopetrotic patients.

## Case presentation

**Figure 1:**
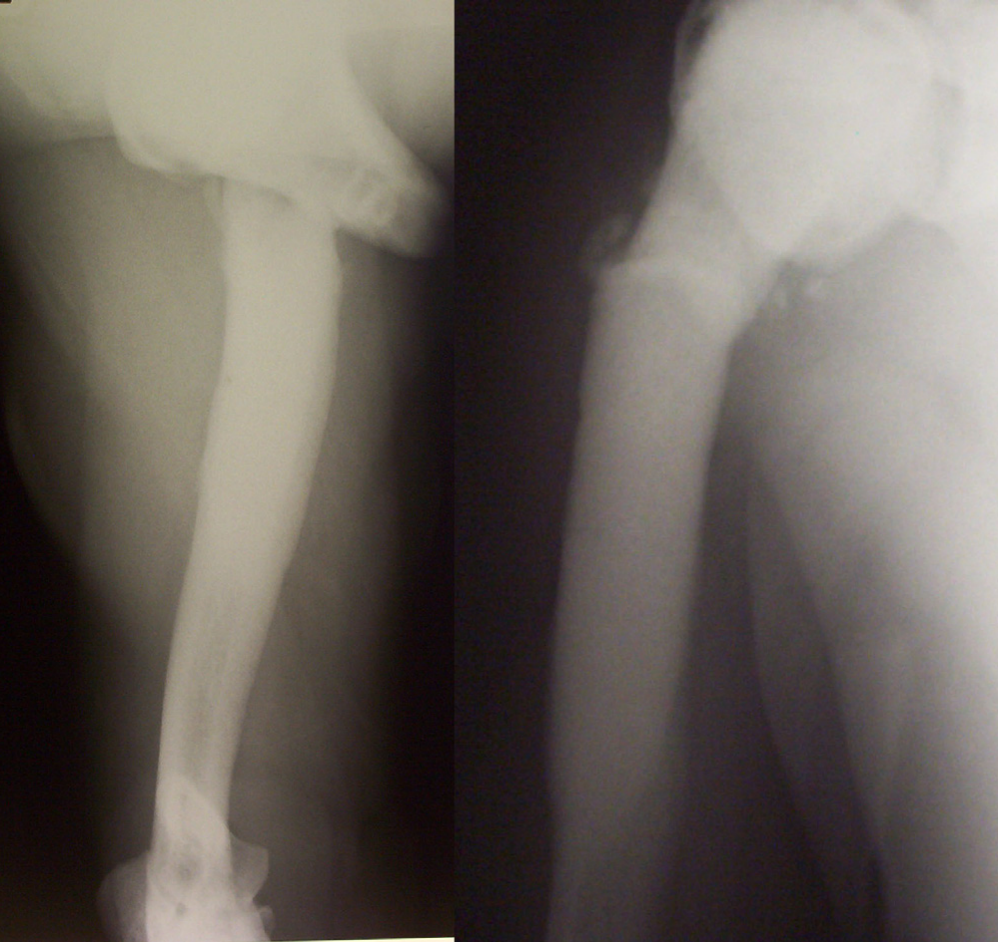
**Pre-operative anterior-posterior/lateral view of non-united fracture of the proximal humerus with osteopetrosis**.

**Figure 2:**
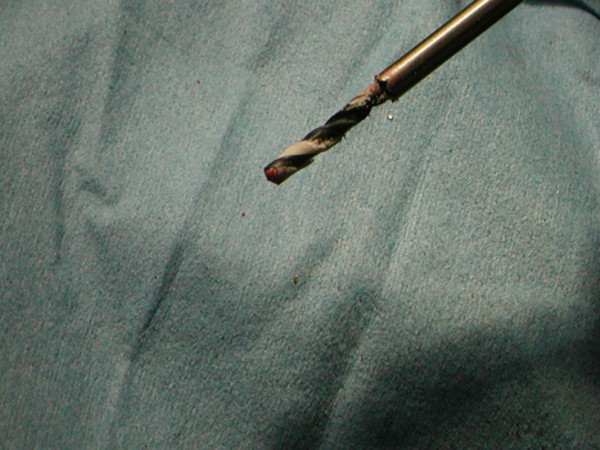
**High-speed steel drill bit used for drilling osteopetrotic sclerosed bone**.

**Figure 3:**
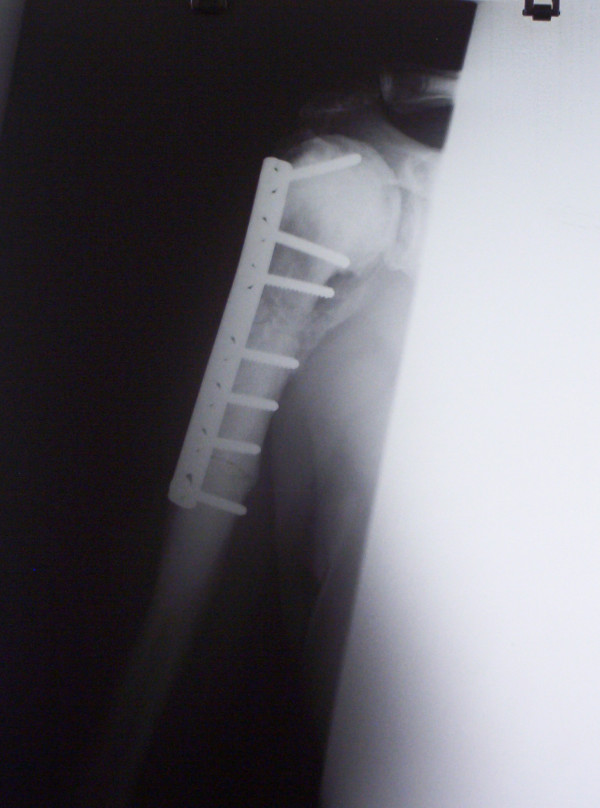
**Postoperative X-ray image of fracture after 6 months with healing and good reduction**.

A 48-year-old male general physician was referred to our unit from a neighbouring hospital with a non-united fracture of the right proximal humerus (Figure 1). The injury was sustained as a result of falling down stairs and was initially managed conservatively for 3 months. There was minimal callus formation with symptoms of fracture union and there was a potential stress line in the distal fragment 4 cm below the fracture. His past history comprised osteopetrosis resulting in fractures of the left femur, left radius and ulna, the latter managed operatively. Open reduction and internal fixation was decided for the fracture of the humerus under general anaesthesia (GA) and axillary block. A delto-pectoral approach was used to expose the fracture. The bone ends were found to be bleeding satisfactorily but there was no medullary canal. Drill holes were inserted for a short distance. The cortex adjacent to the fracture was petalled with osteotome. A 2.5 mm high-speed steel (HSS) drill bit (Synthes, UK) was used with saline cooling. A drill motor with low speed and high torque was used. The drill was frequently removed from the bone to clear the flutes of dense accumulated bone swarf (Figure 2) and saline irrigation was used at all times. The holes were then over-drilled with a standard drill bit to the required 3.2 mm to accept a 4.5 mm screw. A standard 4.5 mm cortical tap was used, frequently reversed and withdrawn for cleaning. A standard 3.2 mm drill was used to attain the right diameter. A plate of sufficient length was used to reach beyond the area of the stress line. It was possible to achieve a secure hold with all the screws. After reduction and fixation, Bone Morphogenic Protein-7 (BMP-7) paste (OP1, Stryker, UK) was applied around the fracture site before closure. The BMP Ossigraft (OP1) was prepared and applied all around the fracture site. A support sling was used for 6 weeks although limited active assisted mobilisation was started on the second postoperative day. After 3 months, there was good evidence of callus formation and fracture healing (Figure 3) along with a full range of motion at the shoulder joint.

## Discussion

Currently, osteopetrosis is considered to be a syndrome with excessive bone density occurring as a result of abnormal function of osteoclasts [3]. Three clinically distinct forms of osteopetrosis have been recognised – the infantile malignant autosomal recessive form, the intermediate autosomal recessive form and the adult benign autosomal dominant form. The disease represents a spectrum of clinical variants because of the heterogeneity of genetic defects resulting in osteoclast dysfunction [4]. The propensity to fracture is seen in all three types but is a major complication in the autosomal dominant form because of the normal life span of patients in this category [5]. Most of the fracture patterns are transverse or short oblique and involve diaphyseal fractures of the long bones of the upper and lower extremities. These can be managed successfully non-operatively especially in children, however time for healing is often prolonged [6,7]. Operative management of diaphyseal fractures is useful for patients where the fractures are recalcitrant to conservative treatment or where there is a risk of developing a disabling deformity, such as with recurrent fractures or pre-existing deformities [6]. Operative fracture management can be technically difficult due to hard brittle bones without a medullary canal. Re-fracture and infection of non-united fractures have been reported after operative management, particularly with screw plate fixation [8]. In order to overcome the technical difficulties regarding drilling and reaming of hard sclerotic bones, recommendations have been made to use high speed electric drill bits, frequently cooling them and clearing the flutes while drilling and using the graduated drill bit system to overcome drill breakage and over-heating [9,10]. However, after internal fixation, implant failure and non-union are still a major risk [6,8]. We successfully overcame this complication by using Bone Morphogenic Protein (BMP) which plays a crucial role in bone formation by stimulating mesenchymal cells and differentiating them into osteoblasts [11]. BMP has proved to be a very good tool because of its osteoinductive property, resulting in good callus formation and healing of fractures.

## Conclusion

Osteopetrosis introduces technical limitations to the traditional treatment of fracture management that may be minimised with specific pre-operative planning. In the treatment of non-united fractures in osteopetrosis, the use of an HSS drill bit along with careful attention to drilling technique can help avoid bit breakage and thermal bone injury that may produce ring sequestrum or destroy the already scant osteogenic cells. BMP-7 may be used as an osteoinductive agent in this situation.

## Abbreviations

BMP: bone morphogenic protein; HSS: high speed steel

## Consent

Written informed consent was obtained from the patient for publication of this case report and any accompanying images. A copy of the written consent is available for review by the Editor-in-Chief of this journal.

## Competing interests

The authors declare that they have no competing interests.

## Authors' contributions

IR and DB conceived the study, participated in its design and coordination and helped to draft the manuscript. JFH and IR conducted the operation. JFH and AK revised the article for intellectual content while IR and AK carried out the literature review and the review of the patient's medical records. All authors read and approved the final manuscript.
